# Home-based rehabilitation experience of children with congenital muscular torticollis: a qualitative study

**DOI:** 10.1186/s12912-025-03360-x

**Published:** 2025-07-01

**Authors:** Youqiong Lu, Lin Mo, Gefang Li, Dan Xiao, Chen Mu

**Affiliations:** 1 Department of Orthopedics Children’s Hospital of Chongqing Medical University, National Clinical Research Center for Child Health and Disorders, Ministry of Education Key Laboratory of Child Development and Disorders, Chongqing Key Laboratory of Structural Birth Defects and Reconstruction, Children’s Hospital of Chongqing Medical University, Chongqing, P.R. China; 2 Outpatient Department Children’s Hospital of Chongqing Medical University, National Clinical Research Center for Child Health and Disorders, Ministry of Education Key Laboratory of Child Development and Disorders, Chongqing Key Laboratory of Structural Birth Defects and Reconstruction, Children’s Hospital of Chongqing Medical University, Chongqing, 401120 P.R. China

**Keywords:** Congenital muscular torticollis, Home-based rehabilitation children, “5E” rehabilitation model, Qualitative research

## Abstract

**Background:**

Early physical therapy holds significant importance for the rehabilitation of children with congenital muscular torticollis. Comprehensive physical therapy combined with home-based care intervention has been developed as well; However, certain issues persist in practical application, such as poor caregiver compliance, inability to adhere to rehabilitation in the long term, lack of disease - related knowledge, etc. Thus, this study aims to offer a reference for the formulation of a home-based rehabilitation program for this population by comprehending the actual experience and requirements of home-based rehabilitation for children with congenital muscular torticollis.

**Methods:**

Through purposive sampling, a descriptive phenomenological study was conducted on family members of children with congenital muscular torticollis from two hospitals in Chongqing Province, China. Semi-structured in-depth interviews were carried out either face-to-face or via video conferencing in the hospital conference room and were evaluated using Colaizzi’s 7-step phenomenological analysis.

**Results:**

A total of 11 primary caregivers of children with congenital muscular torticollis were interviewed, and 9 categories were derived from this study. There were 3 themes for the home-based rehabilitation of children with congenital muscular torticollis: (1) Perceptions and attitudes, (2) Dilemmas, and (3) Trust and need.

**Conclusion:**

The findings indicate that during the home-based rehabilitation process, the primary caregivers of children with congenital muscular torticollis have diverse needs and difficulties. The respondents expressed their hope that medical workers could enhance communication with them, provide professional support, and urge them to adhere to training. Among the numerous needs, the teaching of professional knowledge and skills for home-based rehabilitation training is the most desired.

**Clinical trial number:**

Not applicable.

**Supplementary Information:**

The online version contains supplementary material available at 10.1186/s12912-025-03360-x.

## Introduction

Congenital muscular torticollis (CMT) is a benign muscle disease in children. Recent studies have shown that the incidence rate of CMT has increased significantly, about 0.3-3.92% [1, 2]. About 16% of newborns suffer from CMT [3, 4], and early manual correction treatment within 3 months after birth can achieve a cure rate of up to 90% [5]. If not treated in time, it will cause asymmetry in jaw and facial size, strabismus, and even secondary cervical spondylosis and thoracic scoliosis, seriously affecting the healthy growth of children [6]. Through literature review, it was found that physical therapy and orthopedic tools were mainly selected for treatment in some countries [7, 8]. The clinical practice guideline (CPG) released by the American Physical Therapy Association provided a comprehensive standard of care for the physical therapist [9]. It included five “first choice” interventions: passive cervical range of motion (ROM), active cervical and trunk ROM, facilitation of symmetrical movement, environmental adaptations, and parent education [9]. Passive cervical ROM, or “manual stretching”, was the most common intervention when addressing CMT and had excellent outcomes when performed at high frequency and supplemented with active ROM exercise like prone play. In some countries, rehabilitation is carried out by rehabilitators at home, or the skull fixation orthosis is used for correction, and botulinum toxin type A injection is used for conservative treatment [10]. In China, the treatment of CMT children is roughly the same as the CPG, and supplementary therapeutic methods such as acupuncture, microwave - assisted, acupoint massage, and microcurrent therapy were added. However, there is a huge demand for rehabilitation in China, it is impossible to achieve the recommended high - frequency treatment sessions merely through several hospital - based treatments per week. Therefore, CMT children’s caregivers adherence, as defined in this study as compliance with the home rehabilitation training, is crucial in achieving timely and optimal outcomes.

Some Chinese scholars have proved that home-based rehabilitation training can cultivate children’s activity habits and correct their habitual deviation, which may help improve clinical efficacy and shorten the course of disease [11, 12]. In 2006, home-based intervention was considered to promote the rehabilitation of CMT children, combining usual manual therapy with home-based rehabilitation training can significantly improve the prognosis of CMT children [12, 13]. American scholars encourage caregivers to implement home exercise programs for CMT children in their daily life to improve the recovery rate and shorten the intervention time [11]. However, existing studies only illustrate the importance of home - based rehabilitation for conservative treatment of CMT, with relatively few instructions on the specific content of home - based rehabilitation, and do not pay attention to the inner needs of family rehabilitation implementer, and lacking understanding of the factors hindering home rehabilitation. Therefore, we do not know how caregivers perform home-based rehabilitation? And what are his thoughts and needs about home rehabilitation, and what reason makes him want to give up?

Through the investigation, we found and summarized the following problems in home - based rehabilitation of CMT. Firstly, in other countries, rehabilitation therapists can provide on-site services, but it is impossible for the physical therapist (PT) to meet the recommended high frequency in isolated weekly or biweekly visits. In China, rehabilitation needs are large and therapists are scarce, PT can only go to the hospital, so in clinical practice, many children fail to adhere to rehabilitation. Secondly, with the increase of CMT children’s age, the parents’ compliance is decreased, this is consistent with the study by Audrey Stitt [14]. And prolonging the treatment period increases the risk of surgical intervention [15]. Then, only a few families participate in the rehabilitation treatment model, Some caregivers of CMT children believe that the treatment of therapists is sufficient [12], they do not pay enough attention to home-based rehabilitation [6]. After that, the family members of CMT children lack the knowledge of congenital torticoleste treatment. Caregivers may choose to abandon home rehabilitation due to ignorant or unwillingness and entrust the entire rehabilitation treatment to the therapist [16].Oledzka et al. found that parents of infants with CMT experienced pressure to “correctly implementing the home rehabilitation” in their child’s diagnosis and treatment [17].Finally, the health education provided by medical staff cannot fully meet the rehabilitation needs of them, and the CMT children’s caregivers are not well prepared for home rehabilitation. Some foreign scholars have proved that evidence - based resources are needed to manage the stress associated with CMT diagnosis. Different from chronic diseases, acute diseases such as CMT require time - sensitive approaches and may need more emotional and resource support in the short term [14].

Considering the above mentioned problems in home-based rehabilitation, an in-depth analysis of the experience and needs of home-based rehabilitation for CMT children is very important for the solution of existing problems and future interventions. To date, only Oledzka et al. and Stitt et al. have investigated the parental experience with, and perceptions, a physical therapy course of care for their infant with CMT [14, 17]. Oledzka et al. highlighted parents’ experiences of stress and anxiety relating to the diagnosis and treatment for their infant with CMT, including performance of the home rehabilitation program [17].Their study describes three unique challenges for the parent: correctly implementing the home rehabilitation, time management related to the home rehabilitation, and difficulty breastfeeding [17].Stitt et al. was to describe parent perceptions of the home exercise program for infants with CMT, and how these perceptions evolved over a PT plan of care. While both Oledzka et al. and Stitt et al. identified important factors influencing the parent experience while managing their infant’s CMT, neither study investigated parents’ needs or ideas about the specific evidence-based interventions assigned to them in their home rehabilitation. This is not conducive to the refinement of home-based rehabilitation programs and the real need to achieve feedback in home-based rehabilitation.

Therefore, this study aims to understand the real experience of home rehabilitation training of CMT children in more detail through qualitative methods, to reveal the unrecognized feelings and emotions of children’s caregivers during home rehabilitation training, so as to better analyze the hindering factors and needs of children’s home rehabilitation.

Considering the changes in the rehabilitation process, after group discussion, the “5E” rehabilitation model was selected as theoretical guidance to help caregivers of CMT children establish positive cognition of the disease, promote effective positive coping and maintain rehabilitation behavior.

“5E” Rehabilitation model was developed by Life Options Rehabilitation Advisory Council in the 1994. “5E” rehabilitation model is a comprehensive rehabilitation model that integrates physiology, psychology and society, including five parts: encouragement, education, exercise, employment and evaluation [18]. After years of exploration and practice, this model has gradually adapted to the group characteristics, economic level and medical service model of patients with chronic diseases in China, meeting the needs of China’s medical development. Mainly implemented by nurses and health educators, the “5E” rehabilitation model has been widely developed in China in recent years and has become a common rehabilitation nursing model [19]. Chinese scholars have applied this model to the health education of diseases, and the effect is better than the general model [20, 21].

## Methods

### Design

Semi-structured interviews were used for this descriptive phenomenological study. This study sought to improve our understanding by witnessing first-hand the perspectives of people with experience of home-based rehabilitation. A qualitative descriptive design was used to investigate personal thoughts about home-based rehabilitation in order to discover unrecognized experiences and real rehabilitation needs [22]. CMT children’s “caregivers”, as defined in this study as those who were primarily responsible for home-based rehabilitation training, “family members”,as defined in this study as family members other than the primary caregiver.

### Respondents

In this study, purposive sampling was used to select children diagnosed with CMT and CMT children’s caregivers of two hospitals in Chongqing from October 2022 to January 2023. Based on caregivers’s age, educational levels, socioeconomic backgrounds and severity of the disease in children, the participants were selected for in-depth interviews, following the principle of maximum differentiation. Considering the stability and persistence of home-based rehabilitation, the interviewees selected the main caregivers of the children. However, due to the division of labor of families and practical economic problems in China, almost all children under 1 year old are taken care of by their mothers, so most of the subjects of this study are mothers. Under the guidance of the “5E” rehabilitation model, and combined with literature review and group discussions, semi-structured interviews were conducted with the primary caregivers of CMT children.

Inclusion criteria: clinical diagnosis of CMT; children who were treated conservatively in the outpatient department; family members aged 18–60 years old who were responsible for the daily care of children; family members of the children have normal communication and understanding ability; Participation in this study was voluntary.

### Data collection

A multidisciplinary team consisting of 2 specialist doctors, 2 specialist nurses, 1 rehabilitation nurse, and 1 nursing student was established to form a research team. To acquire data, we conducted semistructured interviews with CMT children’s caregivers. The participants for the guided interviews were chosen to collect information about the home-based rehabilitation training for CMT children. The descriptive phenomenology of qualitative research was used as a guidance, and data was collected through face-to-face, telephone or WeChat video interviews. Before the formal interview, an interview outline was developed through literature review and group discussions. The interview outline was revised through pre-interview with 2 family members. The final version of the interview outline was formed after being reviewed by 1 chief nurse, 1 master of nursing, and 1 specialist doctor. An interview template was developed specifically for this study (see Supplementary Material 1), and the detailed interview outline is shown in Table 1.

**Table 1:** Interview outline for CMT children’s caregivers seeking experience

NO.	Questions
1	Do you know about congenital muscular torticollis? What treatments are available? What is the way to gain knowledge about this disease?
2	What is your perception of home rehabilitation training?
3	What kind of home rehabilitation training have you done for the children, and how did you do it?
4	What doubts and difficulties have you encountered in the process of home-based rehabilitating? How is it solved?
5	What do you consider to be the most crucial aspect of home rehabilitation? What are the most challenging things to adhere to?
6	What do you hope the medical staff can do for you during the rehabilitation training?

The sample size was determined by information saturation. Data saturation occurs when no new information, themes, or codes emerge from subsequent interviews and the data begin to mirror that of earlier interviews. After interviewing 11 caregivers, the responses from caregivers 12 and 13 became repetitive and no new themes emerged, so we considered the data to be saturated and identified 11 as the final interviewers. Eventually, we summarized 9 categories of need information (see “Results” section for details). In order to eliminate the possibility of exposure to new knowledge, the first author conducted two more interviews from December 20, 2023 to December 31, 2023, and still no new themes emerged. Finally, 11 participants were interviewed, and their general information is shown in Table 2. Among the interviewees, we enrolled 11 children along with their primary caregivers. Five children were less than 3 months old (45.5%), eight were only children (72.7%), nine had masses(81.8%). The primary caregivers are almost always mothers(90.9%), ages ranged from 20 to 65 years, with an average age of 33.1 years, six had undergraduate education levels (54.5%). All 11 participants were the main caregivers of CMT children and participated voluntarily.

**Table 2:** General information of children and their primary caregivers (*n* = 11)

NO.	Age in months	Sex	Mass	Primary caregiver	Age	Education levels	Only child
N1	3	Female	Yes	Mother	26	Undergraduate	Yes
N2	2	Male	Yes	Mother	24	Junior college	Yes
N3	4	Female	Yes	Mother	28	Undergraduate	Yes
N4	1	Male	No	Mother	32	Undergraduate	No
N5	2	Male	Yes	Mother	22	Junior college	Yes
N6	3	Female	Yes	Mother	34	Undergraduate	No
N7	7	Female	Yes	Mother	42	Senior high school	No
N8	4	Male	No	Grandma	65	Senior high school	Yes
N9	1	Male	Yes	Mother	34	Undergraduate	Yes
N10	2	Female	Yes	Mother	20	Senior high school	Yes
N11	3	Female	Yes	Mother	37	Undergraduate	Yes

The purpose and reason of the interview were introduced before the interview, and the interview place was chosen in a quiet and comfortable environment. During the interview, the order of questions were adjusted according to the specific situation, and the researcher actively adds open-ended questions and records valuable answers. The interview time was controlled within 60 min. During the recording, the non-verbal expressions of the interviewees, such as expressions, postures and gestures, were observed and recorded. Their viewpoints were clarified and confirmed timely during the interview. When conducting interviews through video, the video equipment were adjusted in advance and conducted under the condition of stable network. The facial expressions and tone of the interviewees were observed and recorded.

### Data analysis

All interview data was anonymous to protect the privacy of the interviewees. Within 24 h after the interview, one researcher listened to the recording repeatedly and transcribed and analyzed the data using a transcription software to improve the research notes and information. Within 24 h after the transcription, another researcher checked the recording and text. The interview data were evaluated using Colaizzi’s 7-step phenomenological data analysis [23]. Colaizzi’s 7-step phenomenological was developed by Paul F.Colaizzi in his book-《Existential-Phenomenological Alternatives for Psychology》,which was Published by Ronald Valle and Mark King in 1978.And the chapter was titled Psychological research as the Phenomenologist views it. It describes in detail that in order to ensure the authenticity, accuracy and credibility of phenomenological research, researchers should follow seven steps to analyze the data they collect. Which is detailed below. ① The two researchers practiced reading and collating raw data by transcribing all responses provided by respondents, including verbal and non-verbal expressions, such as idiomatic expressions, facial expressions, vocal pauses, and sighs. ② The researchers identified and targeted meaningful responses by dismantling, analyzing, reorganizing, and highlighting relevant data. ③ Coding for recurring ideas, theme construction is the process of creating or coding repeated ideas. ④ Using manual coding in combination with the Nvivo11 program, topics were clustered when similar or related codes were put together to generate categories and subcategories. ⑤ The remarks of typical original respondents were included to fully represent the phenomena studied [24]. ⑥ The researchers created the basic framework to include grouping related topics and descriptions, performing iterative comparisons, drawing similar points of view, and developing themes. ⑦ In validating the underlying structure, participants received the topic framework to be validated. Then the framework was organized according to a certain order and theme. If there were any deviations, the researchers would have to start over.

Taking theme 1 as an example: ① Two researchers repeatedly and carefully read the collected data and were fully familiar with all the content provided by CMT children’s caregivers; ② Word by word analysis, identify meaningful statements related to the research question, such as “willing to do rehabilitation training, do not want to do surgery, did massage at home, correction at home” appeared many times. Through disassembly and analysis, these answers all express their wishes.③ Construct meaning units by placing the existing preset hypotheses related to the phenomenon as far as possible. “I did……” in response to the repeated “yes” They were coded as class A and class B respectively. ④ Collecting the opinions of type A and type B after coding, using manual coding in combination with the Nvivo11 program, topics were clustered when similar or related codes were put together: CMT children’s caregivers made many attempts and were very willing to carry out rehabilitation training at home; Themes are grouped together as “attitudes”. ⑤A detailed description of the theme formed in the previous step, and a representative original statement of the respondents was selected; ⑥ The basic framework was created, will be similar to “willing” “at home to do……"do an iterative comparison. To identify and extract similar views, construct a short and meaningful phrase, namely, Perceptions and attitudes; ⑦ The theme and framework will be returned to the respondents for verification, asking whether it accurately captures their real experience. If there is any situation against the wishes of the respondents, the analysis will be started from the first step.

### Researchers’ backgrounds and potential biases

The researchers of this project consist of in-service doctors, nurses and rehabilitation nurses, all of whom possess a certain medical knowledge background. There exists an imbalance in the knowledge dimension compared with the interviewees, and deviations might occur when describing the problem. Secondly, some researchers have expectations for home-based rehabilitation training, which may potentially introduce bias, and they believe that home-based rehabilitation training will undoubtedly promote the rehabilitation of children with congenital muscular torticollis. Most importantly, it is uncertain whether respondents exaggerate the role of medical staff in home training out of fear of being treated differently when their children are in the hospital under the care of medical staff. The researchers were not directly involved in the children’s home rehabilitation training, so we cannot be sure whether these issues had an impact on the respondent’s description. Therefore, it is necessary to implement quality control.

### Quality control

The Respondents were informed that they could withdraw at any time during the interview, and they promised that it would not affect their subsequent treatment, that all the data were only for research purposes, and that the names of the respondents would be replaced by codes, and that the data would be destroyed after the study was over.

The researchers have been engaged in torticollis massage treatment for more than 5 years, and have a good and stable relationship with the participants. In order to reduce the potential bias of rehabilitation personnel, we developed a family rehabilitation program based on the “5E” rehabilitation model, and rehabilitation personnel intervened according to the content of the program. Secondly, our rehabilitation personnel take turns to be responsible for each independent link, and are not responsible for the whole process of any rehabilitation object. This study was supervised by a nursing professor. Before the interview, we fully considered the basic information such as age, gender and education level to recruit representative participants. In addition, the interview content was revised based on a pre-interview and professional discussions to make sure the usability and scientificity of the interview outline. After the transcription, the research team held discussions to ensure the accuracy of the analysis.

To establish credibility, two researchers were invited to process the data. During the data analysis phase, a graduate student who was not involved in the study but was familiar with qualitative research methods was invited to independently read the transcribed data and refine the subjects to ensure the objectivity and authenticity of this study. Two scholars were invited to discuss the obtained topic in detail and the controversial aspects. For example, the problem of medical resources in China: does rehabilitation resources match the needs? Are there possible ways to improve it? Whether it is appropriate for national policies to appear in the text? Finally, we believe that home-based rehabilitation is feasible in china, and the Chinese method can provide reference for foreign countries. All interviewees were asked to confirm the topic results and they all agreed with the results.While working on this manuscript, the Consolidated Criteria for Reporting Qualitative Studies guidelines (COREQ) were followed [25].

### Ethics approval and consent to participate

The Institutional Review Board of Children’s Hospital of Chongqing Medical University approved the study (Ethical approval number: 2022-No.297). All participants signed written informed consent. Each participant was labeled with a number and all collected data was strictly confidential.

## Results

A total of 11 CMT children’s caregivers were interviewed from two children’s hospitals, all families had a stable income,10 were mothers, only 1 was grandma, and 8 had college education or above, with good understanding and execution ability, as presented in Table 2. Three themes and nine subcategories were derived and presented in Table 3. To represent the perspective of CMT children’s caregivers, the following explanations and direct quotes (Chinese translated) describe the meaning of each theme.

**Table 3:** Main categories and subcategories of studies

Main categories	Subcategories
Perceptions and attitudes	Willing to do home-based rehabilitation training
	A lot of effort has been made
Dilemma	Physical and mental exhaustion, lack of family support
	Medical resources are scarce
	Lack of home rehabilitation related knowledge
Trust and Need	Prejudice: Old ideas and short video intrusion
	Think home rehabilitation is not helpful
	Fear of doing rehabilitation at home
	Desire for professional support

### Theme 1: perceptions and attitudes

CMT children’s caregivers had appropriate cognition and positive attitude towards home-based rehabilitation. They were willing to do home rehabilitation and thought they had made an effort.

#### Willing to do home-based rehabilitation training

Some CMT children’s caregivers believed that there were more benefits to not undergoing surgery. In China, some family members believe that non-surgical treatment can provide more employment options for the child, and home rehabilitation can promote the success of treatment. They use home rehabilitation as an adjunct to hospital treatment, eager to accelerate healing and avoid surgery.

*“My child is so young*,* and the operation is too dangerous requiring general anesthesia…We don not want the child to suffer…Therefore*,* we are willing to carry out home-based rehabilitation*,* hoping to shorten the treatment time and improve the effect of treatment outside the hospital.” (N1).*

*“There are scars associated with surgery and some professions (such as professional military personnel*,* some aviation personnel*,* etc.) have restrictions on this. For the sake of my child’s future career choices*,* I will insist on home-based rehabilitation training.” (N9).*

*“I am willing to learn and try everything that is good for baby to cure CMT… Although it will give me very little time to rest…I don’t want her to have surgery.”(N4).*

#### A lot of effort has been made

In the interviews, most caregivers believed that they had made efforts in all aspects of rehabilitation. The rehabilitation training was mainly performed in two aspects: local massage on time and maintaining health-related behaviors. Most of the children’s caregivers were corrected in feeding, sleeping, teasing and head-up training. According to statistics, 80% of the children received at least 2 local massages per day and each lasting more than 5 min. Among the interviewees, N2, N5, N6, N7, and N10 children received regular local hormone(Triamcinolone acetonide) injection to soften stiff connective tissue.

*“I give him a local lump massage at home every morning and afternoon…Put him on his stomach for head-up training…When we going out*,* I also holding he with the affected side to make his neck stretch as much as possible.” (N6).*

*“I am sleeping on his affected side*,* allowing him to stretch the affected side muscle*,* and i massaging the lump locally during my free time… I corrected his inclination while he slept…Entice him to turn his head to the sick side while he drinks and plays…His father cooperate with me to do muscle stretching for him as a bedtime exercise…I think I do my best to improve his condition*,* so as not to leave myself regretful.” (N4).*

*“I chose to inject triamcinolone acetonide intramuscular into the firmer mass*,* hoping for a quick response… As long as the treatment works*,* I’m willing to try.” (N6).*

### Theme 2: dilemma

CMT children’s caregivers think that they face many difficulties in the process of home rehabilitation, and if they can have support from family and society, which would be more conducive to the rapid recovery of the disease.

#### Physical and mental exhaustion, lack of family support

Caring for a sick infant and performing daily rehabilitation exercises can be cumbersome. Lack of family support can make the recovery process even more burdensome.

*“My home is in the suburbs. In order to go to the hospital for treatment every day*,* my children and I rented a house near the hospital alone…But I’m too tired to take care of the children by myself…and I often don’t sleep well…Sometimes I want to give up*,* but I have to work for my children…I was troubled by the psychological changes I suffered.” (N3).*

*“After I retired*,* taking care of my grandchildren became my job…I have to carry milk powder*,* water*,* diapers*,* and take my grandson on public transport every day*,* which is very hard for me…Going to the hospital every day for training is a big challenge for me*,* so i don’t have the energy to rehabilitate her at home.”(N8).*

*“First*,* there was no suitable traction bed at home; Second*,* there was no one at home to help me with my rehabilitation training…Therefore*,* home-based rehabilitation is not possible.” (N5).*

#### Medical resources are scarce

Health care providers are busy, have limited communication time, and medical resources are unevenly distributed, some places do not have the conditions for rehabilitation. Rehabilitation staff did not have the time and energy to educate caregivers about home rehabilitation.

*“There are so few hours in the hospital each day and so many people queuing for treatment…We only have 5–10 min to learn the rehabilitation experience.” (N9).*

*“Doctors and nurses are so busy every day that they have little time to teach me rehabilitation methods.” (N1).*

*“It would be great if we had more therapists guiding us every day…But the reality is that therapists are few and medical staff are busy.” (N4).*

*“My community hospital doesn’t do manual stretching…and they always recommend that I go to a large hospital.”(N3).*

#### Lack of home rehabilitation related knowledge

Most of the respondents mentioned that they had no previous knowledge of home-based rehabilitation training and its treatment for CMT. Almost all interviewees hoped to obtain more professional knowledge related to rehabilitation from medical staff, but the outpatient treatment time was short and medical staff were busy. Health education in hospital and online media can not meet the needs of interviewees for medical information. Several participants shared that they sought out information on the internet and were afraid of the images that they saw. They believed that manual stretching could not be done at home and hoped to be taught by professionals.

*“We only have time to learn from therapists when we are recovering in hospital. So far*,* only learned the local massage… I don’t know of any other home rehabilitation services.” (N1).*

*“When I touched her neck*,* she would cry*,* and I couldn’t tell whether it was due to resistance to manual stretching or pain. I wish someone professional could tell me “Why”…I couldn’t bear the baby to cry*,* So i had no other choice but to take the baby to the hospital for rehabilitation…She would rather do rehab with a therapist than with me.” (N7).*

*“When he trains at home*,* his neck makes a “clacking” sound*,* which makes me too scared to do rehabilitation…After all*,* we are not professional therapists and cannot bear the consequences of rehabilitation accidents.” (N8).*

*“The baby’s neck is so fragile*,* I can’t imagine how I’m going to stretch it…These images shatter my perception…I think this rehabilitation maneuver should be treated with caution…”(N2).*

### Theme 3: trust and need

The interview showed that caregivers of CMT children lack trust in home-based rehabilitation. Most caregivers thought that they needed to learn professional knowledge and receive skill training before they could conduct rehabilitation training at home.

#### Prejudice: outdated ideas and short video intrusion

Among CMT children aged 1 years old and under who received the conservative treatment, the prognosis of children aged 3 months and under is the best. With early diagnosis and physical therapy, the neck can return to normal activity without deformity [26]. Passive traction of the affected side of the sternocleidomastoid muscle is the most widely used early treatment with few side effects and high safety [5]. But some interviewees said that family members thought it was wrong to shake the baby’s head, so the methods described by the therapist were considered not desirable.

*“The baby has no strength in the neck and that’s why his neck looks skewed…The grandparents thought that shaking the baby’s head would make the baby stupid*,* so we decided to wait until the child was slightly older and had strength in the neck before doing home rehabilitation.” (N2).*

*“Now it’s easy to get resources online*,* especially short videos… Every day a different rehabilitation training method is pushed to me. Home-based rehabilitation is also a good option.”(N3).*

*“The parenting video says that shaking your baby’s head too much can affect mental development… There is a risk of shaking syndrome…”(N8).*

#### Home-based rehabilitation is considered not helpful

Some caregivers themselves performed home rehabilitation but did not improve, so they believed that home rehabilitation was not helpful for curing CMT. Some caregivers found that the child struggled and cried less in the hospital than at home, and believed that the child’s rehabilitation cooperation level was higher in the hospital, so the rehabilitation in the hospital was sufficient.

*“We come to the hospital every day for treatment…and my child’s rehabilitation cooperation level was higher in the hospital…My baby struggled and cried less when he was in the hospital for rehabilitation training. There is no need to do rehabilitation at home to strengthen.” (N5).*

*“I have adjusted the baby’s sleeping position and feeding direction according to the therapist*,* but I feel that there is no effect on the rehabilitation training…It was done in vain.” (N2).*

*“I did rotation training*,* no stretching effect…I don’t think home rehabilitation makes sense.” (N11).*

#### Fear of doing rehabilitation at home

Some of the interviewees said that they did not dare to do training at home. After all, they were not professionals. They were afraid of accidents and did not know how to deal with accidents.

*“My child was diagnosed with torticollis very early*,* but at that time*,* she was also diagnosed with hernia…She would cry when she was treated*,* so we did not treat her until after the hernia operation…And we did not systematically learn how to rehabilitation*,* so we did not dare to exercise at home…On the other hand*,* it was also because of the fear that training may lead to the recurrence of hernia…Her crying makes me nervous.” (N7).*

*“I only have one son and I only have one grandson. They are my most precious people…I dare not do training at home for fear of encountering accidents.” (N8).*

*“It is a training for the baby’s neck…We do not know how to deal with complications and accidents that occur during home-based rehabilitation…I was very resistant…rehabilitation at home is a terrible thing.” (N10).*

#### Desire for professional support

Professional support refers to providing professional knowledge, skills, and resources to help individuals or organizations achieve their goals or solve specific problems in a specific area. Throughout the interviews, almost all interviewees expressed a strong desire for professional support. The interview results showed that most parents recognized the importance of home-based rehabilitation in conservative treatment, but due to the lack of knowledge of home-based rehabilitation training, many caregivers did not know what training to do and how to do it. Even afraid to do rehabilitation at home. Compared with therapists, caregivers gave more emotional responses to babies’ crying. The baby’s intense struggle and crying may cause the caregiver to temporarily give up on recovery, so the baby learns to cry to resist home recovery, but parents cannot distinguish pain from it. They were desperate for help.

*“It is a simple idea*,* because I love my child…So I want to learn how to rehabilitation*,* hoping to help her recover soon.” (N7).*

*“If I learn to train at home*,* I will not have to go back and forth between hospital and home…I will have my own training plan.” (N3).*

*“I can only do a local massage on the lump and prone head-up training… If anyone can tell me more feasible methods*,* I will be very grateful to him.” (N1).*

*“My child is extremely uncooperative when training at home…Whenever he make noise or cough*,* I am afraid and have to stop exercising…I don’t know what situation is normal and what is dangerous…No one teaches me and I do not know if it is normal.” (N10).*

## Discussion

Through a semi-structured in-depth interview, this study showed the cognition and attitudes, dilemmas, trust and needs of CMT children’s caregivers. These open-ended responses complemented the gaps in the quantitative study. Through interviews with CMT children’s caregivers, we understood the significance of establishing a home-based rehabilitation training program, which can help CMT children’s caregivers better adapt to training, shorten the treatment time, and contribute to the recovery of children. According to the guidance of the 5E rehabilitation model, we divided it into the following five parts.

### Encouragement: building confidence and guiding family members to learn actively and persist in training

Research has shown that if the intervention commences after one month of birth, the treatment duration is approximately six months. However, if the intervention begins after six months of birth, the treatment duration might increase to 9–10 months, and a small number of children may fail to restore the normal range of motion of their joints [5]. If conservative treatment proves ineffective, surgical treatment is necessary [27]. Although early conservative treatment is efficacious, it requires long-term and regular adherence to achieve satisfactory outcomes, so it is particularly crucial to assist the caregivers of CMT children in persisting with home-based rehabilitation. In this study, interviews with caregivers of CMT children revealed that participants felt stress about common exercises for CMT family rehabilitation, which is consistent with the findings of Oledzka et al. [17]. In some families, due to the absence of other family members, caregivers feel tired of frequent visits to the hospital for rehabilitation. Or they did not have enough time and energy for home rehabilitation after outpatient rehabilitation, or they gave up home rehabilitation because the child was not cooperative, these perceptions revealed the hesitancy of caregivers to conduct home rehabilitation with the infant. In addition, we identified sources of caregiver stress or factors that promoted caregiver adherence to home-based rehabilitation. Factors that enhance confidence in CMT rehabilitation include early participation in rehabilitation and more professional. Conversely, disadvantages such as exhaustion, lack of knowledge of correct rehabilitation methods, and dealing with rehabilitation accidents were barriers to home-based rehabilitation.

We can support caregivers in the following ways. ①Healthcare professionals can listen more to the inner feelings of caregivers, do not rush to give advice, and give more emotional confirmation, such as “I understand how you are feeling” or “this must be difficult”, etc. Help the caregiver release emotions through listening and dialogue so that the caregiver feels understood and supported. ②Successful cases were actively introduced to build confidence for caregivers of CMT children. Peer support projects should be implemented, and rehabilitation WeChat contact groups can be established to create an online communication platform for the caregivers of children. We should encourage the caregivers of CMT children to actively share the skills and experience of home-based rehabilitation at home, and to share how family members of children make full use of general household tools for rehabilitation training. In the process of implementation, we found that peer support group and wechat contact groups could improve the confidence of caregivers in home rehabilitation. Caregivers who encouraged each other were indeed easier to adhere to, and they could get home rehabilitation experience different from that of rehabilitation doctors. However, there are also drawbacks in the implementation process, such as the standardization and safety of home-based rehabilitation experience is not ensured, different caregivers have inconsistent understandings of the experience, and babies have different reactions to the same rehabilitation experience, which will further complicate home-based rehabilitation. Secondly, the pressure to recover from peer success, the negative impact of peer abandonment of recovery, etc., are all possibilities. ③Advocacy the family-centered holistic care model [28]can help guide all family members to participate in the family training and rehabilitation training of children, encourage family members to express their genuine needs, and affirm and praise their progress. ④Nurses can assist caregivers of CMT children to connect community resources when therapists are busy.

### Education: strengthening guidance and correcting caregivers’ cognition and attitude towards home-based rehabilitation

This interview discovered that the majority of caregivers for children are deficient in disease-related knowledge and professional support, and have no idea about how to carry out home-based rehabilitation training, which aligns with the results of Wang X et.al’s study [12]. Parents lack confidence in manual stretching skills due to fear of doing it “for” rather than “with” their children. In the current rehabilitation model, due to lack of cognition, family members frequently do not participate in rehabilitation, and therapists fail to effectively convey the specific content of home-based rehabilitation to the caregivers of CMT children, which might result in most family members of children not attaching sufficient importance to home-based rehabilitation [29]. Some caregivers believe that home rehabilitation is pointless because of the crying and struggle of their infants. Child caregivers are the core of home-based rehabilitation. Due to various reasons and problems, caregivers lack professional knowledge or are unable to provide sufficient training for children, which may be the reasons that home-based rehabilitation cannot be implemented [6]. As children grow older, their compliance with manual traction therapy deteriorates, thus influencing the treatment outcome and reducing parents’ compliance. In order to avoid prolonged treatment period, making early intervention is necessary for CMT [13].

Scientific guidance to strengthen the knowledge and skills of home-based rehabilitation is necessary. When providing rehabilitation for CMT children, we can offer sufficient home-based rehabilitation information support and training guidance for caregivers through face-to-face communication, brochures, video communication, and WeChat. During the implementation, we found that face-to-face communication with caregivers and on-site training were the best choices, and we could find problems while guiding them. However, because of COVID-19 or busyness, on-site training sessions can not be performed well every time. Rabino et al. found that a parent’s perception of the threat of their infant’s CMT increased the likelihood that they remained adherent to performing the home rehabilitation and attending PT sessions [30]. So we can inform about the possible adverse consequences of CMT, discussing the complications and sequelae of the disease with parents can enhance their compliance with rehabilitation treatment [30]. Secondly, actively impart home-based rehabilitation knowledge and skills, such as muscle anatomy, treatment approaches, living habits (feeding, hugging, sleeping, seducing), local massage, manual pulling, head-up training, eye tracking training, and barthel ball training. Suprenant et al. [31] also affirmed that under strict supervision, parents’ rehabilitation training for their children can achieve the same level of effectiveness as that of therapists.

### Exercise: strengthening rehabilitation skills and improving home-based rehabilitation ability and accident handling

Almost all participants in this study expressed not knowing how to properly perform rehabilitation training at home. With the increase of children’s age, some caregivers also expressed children’s resistance to home rehabilitation, caregivers’ compliance with manual stretching became worse [32]. Stitt et al. found in their study that specific planning and clear communication can enhance parental compliance and confidence in implementing home rehabilitation for their baby. Hence, it is necessary to provide caregivers with home-based rehabilitation programs and guide them in rehabilitation knowledge and skills.

We summarized the common rehabilitation training methods for CMT children and conducted regular on-site training: ①Local massage should be performed thrice a day at home, with the pulp of the index finger and middle finger on the affected side of the sternocleidomastoid muscle mass undergoing obvious kneading for 15 min each time. ②During each manual traction, an attempt should be made to reach the full traction range (lateral flexion of 70 degrees and rotation of 90 degrees) and hold for 10–15 s, with a 5–10 s break in the middle [5]. Each group should be pulled 10 times. CMT children should complete 5 or 10 sets daily. ③Regarding living habits, the child’s head and arms should be turned to the affected side during eating [33]. Rice bags should be placed on both sides of the head to maintain the head and trunk in a neutral position when sleeping. The head should be turned to the affected side when playing [12]. ④During the head up training, parents should fix the elbows and shoulders of the child, ensuring that the elbows are of the same width as the shoulders, and the forearm is supported to encourage the child to look up forward or to the affected side [32]. ⑤For barthel ball training, the child should lie on the barthel ball. Parents assist in fixing their child’s buttocks on both sides, pushing the barthel ball forward to move the child’s chest away from the ball. Another parent holds a toy to induce the child’s head towards the affected side. When the child is fatigued, he can lie on the barthel ball again for rest.

Home-based rehabilitation adheres to the purpose of promoting symmetrical neck extension and head up, and all actions are focused on making the child’s head bend to the healthy side and rotate to the affected side [34]. Facing the fear and pressure of manual traction among caregivers during home-based rehabilitation, family members should be instructed on how to prevent and handle accidents. When doing manual stretching at home, caregivers attention should be paid to observing children’s facial expressions to prevent incidents such as vomiting, choking, asphyxia, cervical spine injury, cervical vascular injury, and muscle fracture. Parents should be taught to master the basic process of handling accidents: immediately stop training upon an accident; assess the pulse, breathing, and consciousness; If there is no breathing or pulse, cardiopulmonary resuscitation should be carried out immediately. Negative pressure suction or artificial respiration was performed, and an ambulance was called. If there is pulse and breathing, the child should be placed in a lateral position immediately and vomit and respiratory sputum should be cleared. Patients with cervical spine injuries should avoid movement, and those with vascular and muscle injuries should receive immediate local compression treatment.

### Employment: developing a balanced plan to maintain the harmonious coexistence of work and training

As maternity leave ends, parents’ priorities shift to balancing work, life, and parenting, and these stresses may reduce adherence to overall recovery. A balanced home-based rehabilitation training plan should be formulated according to the nature of work and rest time, such as rehabilitation training for children by grandparents in the morning, and rehabilitation training for children by parents in the afternoon and evening. Family members should participate and complete the training together, enabling CMT children and their caregivers to adapt to rehabilitation training without affecting their rhythm of life. After primary caregiver returning to social work, home-based rehabilitation can be continued for children.

### Evaluation: establishing rehabilitation files and paying attention to the evaluation of individual factors

The entire process of outpatient conservative treatment should be coordinated with home rehabilitation training. A WeChat mini-program can be established for clocking in, and for recording and evaluating the frequency of rehabilitation training and the rehabilitation awareness of caregivers of CMT children. The overall situation of the child’s family should be assessed, encompassing the child’s family environment, psychological, physiological, and health-related behaviors. Specifically, the family environment may comprise the natural environment, family setting, and social environment. The psychological domain may include peer support and rehabilitation consciousness. The physiological domain may involve age, location, mass or not, neck muscle, and activity. The health-related behavior domain may consist of daily feeding, cuddling, sleeping, teasing, and the frequency of home-based rehabilitation. Based on the evaluation results, individualized guidance can be provided to enhance the caregivers’ cognition and the effect of home-based rehabilitation. We propose the establishment of training files to track and record the whole process of CMT children’s rehabilitation and supervise the implementation of various tasks. We suggest that at the second, fourth, and sixth months after the start of rehabilitation, the Clinical Disease Cure and Improvement Criteria [35], neck passive joint range of motion, and outpatient B-ultrasound results should be used to evaluate the cure of children.

## Limitations and prospects

The selected sample was merely the key caregivers of CMT children in Chongqing Province, China, and was mainly in two hospital settings. Therapists in different environments (such as communities and clinics) or different regions (outside Chongqing province) have differences in techniques, physical therapy, rehabilitation methods, training standards, etc. Consequently, a larger and more representative sample survey is requisite to seek feedback on the home-based rehabilitation experience. In China, children younger than 1 year old are mainly cared for by the mother, and the father is the breadwinner. For the authenticity of the data, the interviewees in this study were the main caregivers of CMT children. In the future, more extensive participants (such as fathers or other caregivers, therapists, doctors, etc.) can be included to offer additional perspectives and enhance the richness of the data.Hence, the results of this study have certain limitations. Future multi-role participants and multi-regional studies are recommended to make the results more representative.

## Conclusions

Through phenomenological methods in qualitative research, in-depth interviews were conducted with 11 main caregivers of CMT children. We identified some problems of CMT children’s caregivers in home rehabilitation. Firstly, it was discovered that CMT children’s caregivers made efforts in home-based rehabilitation training, such as local massage, maintaining rehabilitation behavior, but there were still numerous issues. Additionally, CMT children’s caregivers encounter the predicament of a lack of family and social support. But most parents of CMT children choose to trust medical staff and are eager to obtain assistance. Finally, caregivers of CMT children demonstrate a deficiency in knowledge and skills about home-based rehabilitation, a subjective bias toward stretch therapy and have fears or consider that home-based rehabilitation is unnecessary. This study provides suggestions and ideas for home rehabilitation of children with congenital muscular torticollis, and provides appropriate guidance and support for the formulation of home rehabilitation programs. Further research is needed to determine whether home-based rehabilitation brings different levels of negative emotions and psychological stress to caregivers and to validate the applicability of the rehabilitation strategies presented in this manuscript.

## Electronic supplementary material

Below is the link to the electronic supplementary material.

Supplementary Material 1

## Data Availability

The data presented in this article cannot be shared as the participants did not consent to sharing their responses.Requests regarding the datasets should be directed to the first author.

## Abbreviations

CMT: Congenital muscular torticollis
CPG: Clinical practice guideline
ROM: Range of motion
PT: Physical therapy or physical therapist
