# Supplementary material for: Home-based rehabilitation experience of children with congenital muscular torticollis: a qualitative study
Source: BMC Nurs. 2025 Jul 1;24:758. doi: 10.1186/s12912-025-03360-x (PMC12210643; doi:10.1186/s12912-025-03360-x)
Supplement: Supplementary file 1 — Supplementary Material 1 [file 12912_2025_3360_MOESM1_ESM.docx]

**Interview Template/Questions for Parent Participant**

1.Do you know about congenital muscular torticollis? What treatments are available? What is the way to gain knowledge about this disease?

2.What is your perception of home rehabilitation training?

3.What kind of home rehabilitation training have you done for the children, and how did you do it?

4.What doubts and difficulties have you encountered in the process of home-based rehabilitating? How is it solved?

5.What do you consider to be the most crucial aspect of home rehabilitation? What are the most challenging things to adhere to?

6.What do you hope the medical staff can do for you during the home rehabilitation training?

**Section 1 -- 1 ^st^ interview only.**

I have some background questions for you:

a. What is (Baby’s) current age?

b. At what age did (Baby) get a diagnosis of CMT?

c. Do you know what CMT is? How dou you know?

d. At what age did (Baby) begin PT?

e. How often is (Baby) getting PT?

f. Is this your first child?

g:What do you know about home-based rehabilitation?

**Section 2 (20 min): Interview template**

We're going to talk about your thoughts and needs for home rehabilitation.

| Exercise Name  &/or Activity  Description | Approximate  length of time completed each time (minutes)  And # days  completed  each week | Doubt &/or  difficulty | The most  important  &/or  challenging | Comments:  (1)Thoughts  (2)Feelings  (3)Barriers / difficulties / stress /coping with  (4)Hand placement?  (5)I did not do this one because…or I did this one more because…  (6)Did your partner assist with these?  (7)What do you expect the medical staff to do?  If offers info –“tell me more about that” |
| --- | --- | --- | --- | --- |
| manual stretching |  |  |  |  |
| Head up training |  |  |  |  |
| Massage |  |  |  |  |
| Correction |  |  |  |  |
| Other | Do you feel that you are handling your baby in a way that's comfortable for her/him.  Do you feel confident in your ability to help baby move into different positions  comfortably?  Page 1 of 2 Revision: October 20, 2022  Do babies cry?  Can you tell when baby needs a break? How?  How much help do you have at home?  What would you want new parents to know about this condition? What does it feel like to  have a baby with this health condition? Any a-ha moments you’d like to share?  Thank you for your time. A member of the research team will contact you next month. | | | |

Page 2 of 2 Revision: October 20, 2022
